# Chemistry and Biological Activities of Marine Polysaccharides: Chitin, Chitosan, Chitooligosaccharide, and Their Derivatives

**DOI:** 10.3390/md24100366

**Published:** 2026-10-19

**Authors:** Xiaobo Wang, Min Lv, Hui Xu

**Affiliations:** 1 School of Marine Sciences, Ningbo University, Ningbo 315832, China; 2511130276@nbu.edu.cn; 2 College of Plant Protection, Northwest A&F University, Xianyang 712100, China

**Keywords:** chitin, marine polysaccharide, biological activity, structural modification, mechanism of action, structure–activity relationship

## Abstract

Chitin, mainly derived from marine organisms, is the second-most abundant natural polysaccharide. Notably, marine sponge-derived chitin has recently emerged as a highly promising resource due to its unique naturally pre-organized three-dimensional nanofibrous network and high purity, offering unparalleled advantages for modern biomedical engineering and advanced materials science. Generally, chitin and its derivatives have shown great application potential in the fields of biomedicine and agriculture due to their excellent biocompatibility and diverse biological activities such as antibacterial, anti-inflammatory, immunomodulatory, and antitumor properties. However, the poor solubility and structural inertia of natural chitin limit its development and utilization to a certain extent. In this review, we systemically summarized the biological activities and structural functionalization strategies of chitin, chitosan, chitooligosaccharide and their derivatives from 2020 to 2025. Then, their mechanisms of action and structure–activity relationships (SARs) were concluded. Moreover, the applications of these marine-derived biopolymers in future therapeutics and sustainable smart materials were prospected. Finally, we analyzed the current scientific challenges from laboratory research and development to large-scale application. Hopefully, we enthusiastically anticipate that this review will offer readers valuable insights and proposals on developing chitin-based effective agents in the future.

## 1. Introduction

**Figure 1:**
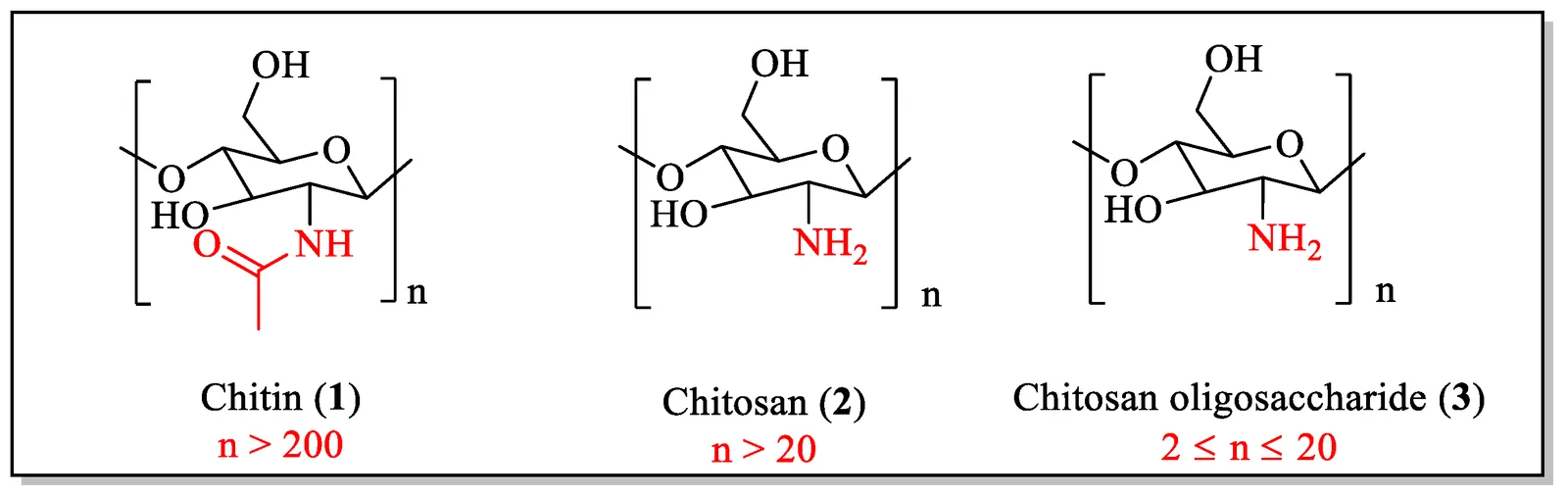
The chemical structures of chitin (**1**), chitosan (**2**), and chitosan oligosaccharide (**3**).

**Figure 2:**
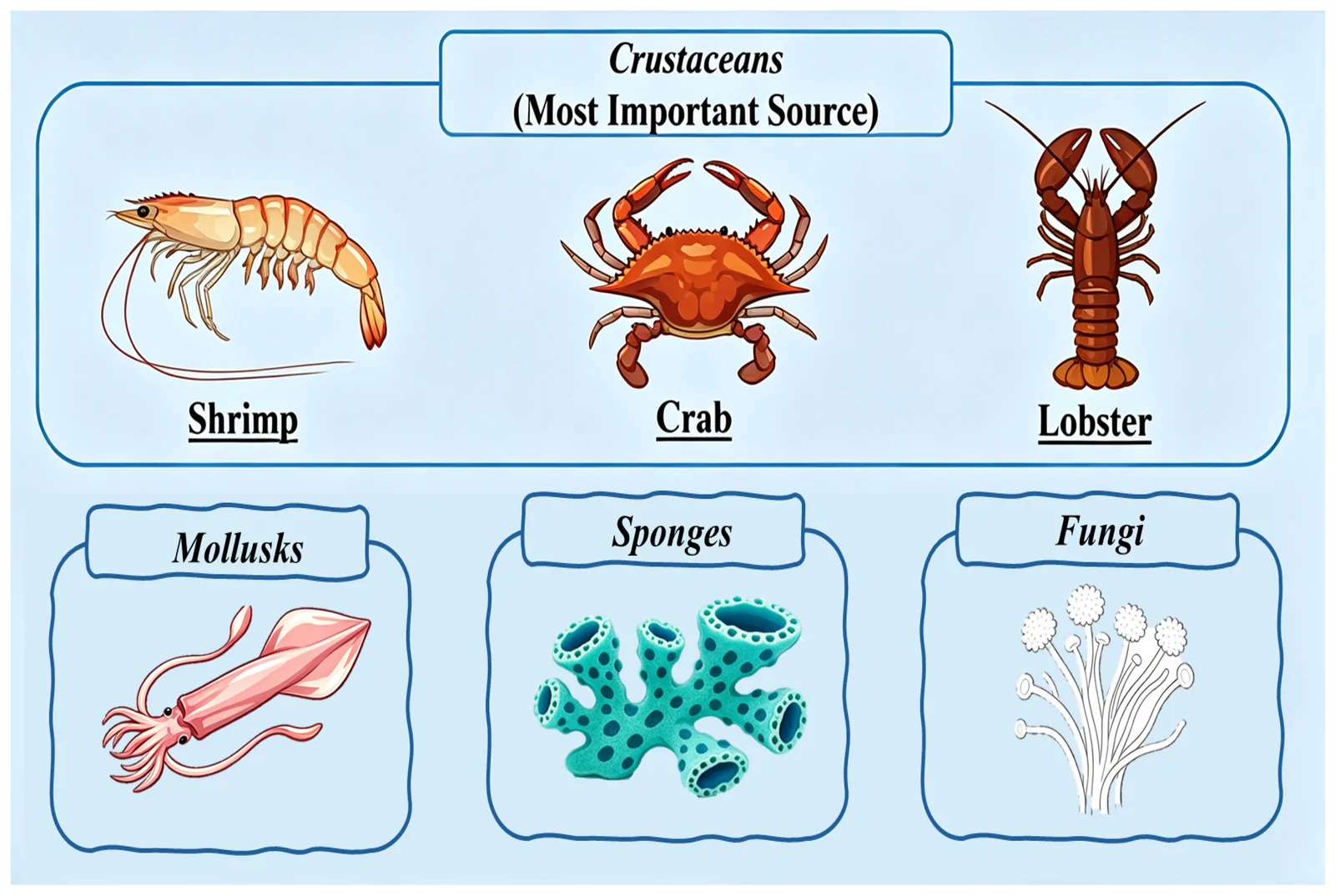
Primary marine sources of chitin (**1**).

Chitin (CT, **1**, Figure 1) is a linear polysaccharide constructed by *N*-acetyl-D-glucosamine (GlcNAc) linked by β-(1,4)-glycosidic bonds. Its natural reserves are second only to cellulose, and its annual biosynthesis is about 10^11–12^ tons. It is the core skeleton component for constructing marine crustacean exoskeletons and fungal cell walls [1,2,3,4]. In addition, it is worth mentioning that marine sponges (especially *Demospongiae*) are also an important and ancient source of chitin [5,6]. The marine sources of CT are shown in Figure 2. In nature, α-CT is the most important configuration, and its molecular chains are anti-parallel and tightly packed, forming a highly ordered crystal structure and complex intermolecular hydrogen bond network. Compared with CT derived from crustaceans, from a crystallographic point of view, sponge-derived CT usually belongs to α-CT with higher crystallinity, and its microcrystalline size and lattice stability are significantly better than those of common arthropod sources. This unique structure endows CT with excellent mechanical strength and chemical stability, but it also leads to its poor solubility in water and most organic solvents [7,8,9,10,11,12]. Therefore, the process of extracting powdered CT from shrimp and crab shells in industry not only involves the violent reaction of strong acid and alkali but also completely destroys the original natural structure of the organism. Unlike crustacean CT, morphologically, sponge-derived CT exhibits an extremely rare natural nanofiber interwoven structure [13]. Without any physical reconstruction such as artificial crosslinking or electrospinning, it can maintain the inherent highly interconnected and macroporous three-dimensional (3D) network skeleton. This naturally preformed 3D scaffold is highly similar to human extracellular matrix (ECM) and does not require complex secondary processing such as electrospinning or 3D printing. It directly provides an ideal platform for the next generation of biomedical and bionic applications [14,15,16,17]. Sponge-derived CT can be converted into chitosan (CS, **2**, Figure 1) by deacetylation treatment. At the same time, the high-density amino and hydroxyl active groups on CS provide sufficient action sites for subsequent chemical grafting, crosslinking and polypeptide modification. Further chemical modification such as carboxymethylation, quaternization or grafting functional groups can also significantly improve its solubility, biological activity and targeting ability, and greatly expand its application in drug delivery systems [18,19].

In fact, the biological activity of CS is not determined by a single structural parameter but is the result of the synergistic effects of degree of deacetylation (DD), degree of acetylation (DA), molecular weight (MW) and substitution mode. Among them, DD determines the cationic charge density and basic physical and chemical properties of chitosan, MW determines its molecular chain flexibility, cell uptake and in vivo distribution, and the substitution mode of chemical modification realizes the directional regulation and functional expansion of activity [20,21,22]. It can be divided into high-MW CS (>300 kDa), medium-MW CS (50–300 kDa), low-MW CS (10–50 kDa), and chitooligosaccharide (<10 kDa, COS, **3**, Figure 1; degree of polymerization (DP): 2–20). MW mainly regulates its biological activity by affecting the water solubility, molecular chain flexibility, endocytosis, lysosomal escape and in vivo distribution of chitosan [23,24]. In addition, the pattern of acetylation (PA) is the third core structural parameter that regulates the biological activity of CT derivatives independent of molecular weight and degree of deacetylation. The PA characteristics of CS and COS are directly determined by the preparation process. The antimicrobial activity of CT derivatives is mainly based on nonspecific electrostatic interactions between protonated amino groups and microbial cell membranes [25,26,27].

In order to improve its processing performance and explore its biological activity, the preparation of CS and COS by chemical or enzymatic deacetylation has become the mainstream strategy. Among them, acidic chitinases with high temperature resistance and high degradation efficiency provide new options for green enzymatic preparation [28]. CS is a product of partial deacetylation of CT, which introduces a highly reactive free amino group, giving it excellent biocompatibility and biodegradability [13]. However, due to the limitation of solubility in a physiological pH environment, further degradation to prepare COS has become a research hotspot [29,30]. COS not only retains the unique natural properties but also shows significant structure–activity relationship (SAR) advantages in the fields of antibacterial, anti-inflammatory, immune regulation, and anti-tumor applications, due to their superior water solubility and cell affinity [31,32].

At present, the research paradigm of CT is changing from single resource extraction to fine chemical modification, enzyme engineering, and physical field-assisted techniques [33,34,35,36]. This paper systematically reviews the latest progress on the biological activities, structural modification, SARs, and mechanisms of action of CT and its derivatives, focusing on the key breakthroughs and future challenges in the transformation from basic research to agricultural and biomedical industries.

## 2. Biological Activities of Chitin, Chitosan, Chitooligosaccharide, and Their Derivatives

### 2.1. Antibacterial Activity

In the family of CT derivatives, COS derivatives are regarded as a new generation of natural antibacterial agents with great development value due to their unique cationic properties and excellent water solubility. COS has a significant inhibitory effect on a variety of Gram-negative and -positive bacteria, including *Escherichia coli* and *Staphylococcus aureus*. The minimum inhibitory concentrations (MICs) of COS against the above two bacteria were 1.25% and 1.85%, respectively [37]. In addition, even *N*-acetyl COS with a low degree of polymerization (DP: 2–3) has specific activity against *Bacillus subtilis* WB600 and *Xanthomonas* [38]. On *Aspergillus fumigatus* and *Aspergillus flavus*, the MIC values of COS were 15.6 and 31.2 mg/mL, respectively [39]. The current consensus tends to believe that MW is the primary factor affecting their antibacterial efficacy [40]. The specific ratio of the highly acetylated sequence to the free amino group is essential for destroying the integrity of the bacterial cell membrane [41]; on the other hand, COS with a low acetylation level (high deacetylation degree) is more efficient in reducing bacterial load [20]. It suggests that the regulation of acetyl groups on antibacterial activity may not be a simple linear relationship but is complexly regulated by various factors such as environmental pH, cell wall structure of the target strain, and COS molecular chain conformation.

The core correlation between DD and the antibacterial activity of CS lies in the cationic charge density. The inhibition time of CS with a DA of 8–12% (DD: 88–92%) on the growth of *S. aureus* and *B. subtilis* was more than 85 h, while the inhibition time of CS with a DA of 35–61% (DD: 39–65%) was only 5–30 h. For plant pathogenic fungi, the mycelial growth inhibition effect of CS with a high DD was also significantly better than that of low-DD samples. However, there was a critical value and strain specificity in this correlation: when DD exceeded 90%, the crystallinity of CS increased significantly and the water solubility decreased, the molecular chain could not fully stretch and bind to the surface of bacteria, and the corresponding antibacterial activity decreased instead. COS with a medium DD (70–85%), which achieves the optimal balance between water solubility and cationic charge density, is the preferred range for most antibacterial applications. In addition, DD had a more significant effect on the activity of Gram-positive bacteria, when compared with Gram-negative bacteria [23,24,25].

### 2.2. Antioxidant Activity

Reactive oxygen species (ROS) are inevitable products of aerobic metabolism in organisms [42]. However, when the body is stimulated by external stimuli, excessive accumulation of ROS and free radicals will break the redox balance and trigger oxidative stress. For natural extracts, such as CT from crickets (*Acheta domestica*), the DPPH scavenging rate is usually distributed in the range of 10–60%, and the FRAP reduction ability is positively correlated with the concentration [43]. However, due to the high crystallinity and low solubility of natural CT, its active sites are difficult to be fully exposed. In contrast, chemical modification can effectively break through this limitation. For example, the removal rate of CT chloroacetate prepared by introducing chloroacetyl through esterification reaction was increased to 56.95% [44], indicating that side chain modification was helpful to improve the electron cloud distribution and reactivity of molecular chains [45]. In terms of enzymatic hydrolysis strategy, low-molecular-weight COS showed superior performance. The comparative study found that the activity of ECOS prepared by the optimized enzyme production–adsorption–enzymatic hydrolysis–product separation system was significantly better than that of commercially available COS. At 1 mg/mL, the clearance rate of ECOS was as high as 92.71%, which was equivalent to the level of vitamin C, while the activity of commercially available COS was significantly lower [46].

### 2.3. Anti-Inflammatory Activity

Inflammation is a defensive biological response of the body to exogenous stimuli such as physical damage and pathogen infection. However, long-term or excessive inflammatory responses can lead to oxidative stress and tissue damage, which can induce a variety of chronic diseases [47]. Asad et al. conducted a systematic pharmacological evaluation of CT derived from *Periplaneta americana* and *Termite* exoskeleton [48]. In the acute inflammation model of xylene-induced ear swelling in mice, CT showed excellent anti-edema activity. In particular, the high-concentration group (500 μL/mL) showed a 46.16% inflammation inhibition rate, which was statistically superior to the standard treatment group of non-steroidal anti-inflammatory drugs (NSAIDs), diclofenac sodium (41.94%). At the molecular level, it is important to elucidate the regulation of structural parameters on anti-inflammatory activity. Using a lipopolysaccharide (LPS)-stimulated RAW264.7 macrophage model (a classic in vitro inflammation screening platform), the researchers explored the intrinsic relationship between DP of COS and immunomodulatory activity. The results showed that the inhibitory effect of COS on the key pro-inflammatory mediator nitric oxide (NO) was strictly dependent on the DP as the length of the sugar chain increased (from D2 to D6) [49]. In addition to macrophage-mediated inflammatory responses, COS also shows a regulatory effect on IgE-induced allergic inflammation. Low-MW COS can inhibit the degranulation of mast cells and the release of inflammatory cytokines in RBL-2H3 cells, and it can alleviate airway inflammation in asthmatic mice, expanding its application potential in allergic diseases [50,51].

### 2.4. Anti-Tumor Activity

Although modern medicine has made great progress in tumor treatment, the invasive metastasis and multidrug resistance (MDR) of malignant tumors are still the main clinical challenges. CT and its derivatives have both anti-inflammatory and anti-tumor pleiotropic effects [52]. COS and its degradation products could exert anti-tumor effects by interfering with the energy metabolism of tumor cells [53]. Because of its unique positive charge surface, CT and its refined derivatives (such as COS) easily interact with the negative charge groups on the surface of tumor cells by electrostatic interaction, which then achieve precise treatment through immune activation or construction of an intelligent drug delivery system (DDS) [54,55,56]. In the field of nanomedicine, COS can be often used as nano drug delivery system to improve the pharmacokinetic behavior of drugs. Hu et al. constructed a COS-modified osimertinib-PLGA nano-delivery system (AZD-PLGA-COS), which could potentially enhance drug efficacy by activating the mitochondrial apoptotic pathway. In the A549 non-small cell lung cancer model, the paclitaxel–COS liposome group achieved a tumor inhibition rate of up to 86.4%. This significant effect is attributed to the enhanced penetration and retention of liposomes in tumor tissues (EPR effect) by COS modification, which provides strong empirical support for the development of the next generation of anti-tumor nano-preparations [55,56]. Elemental functionalization of COS can give them stronger targeted killing ability. For example, the seleno-chitosan oligosaccharides derivative (COS-Se) designed by Jiang et al. showed dual efficacy of anti-angiogenesis and anti-metastasis in the treatment of gastric cancer [57].

The biological activities of COS derivatives are summarized in Table 1. Chemical modification of COS does indeed result in changes in their structural characteristics and physicochemical properties, thereby enhancing or altering their biological activities, such as antibacterial, antioxidant, anti-inflammatory, and anti-tumor activities.

**Figure 3:**
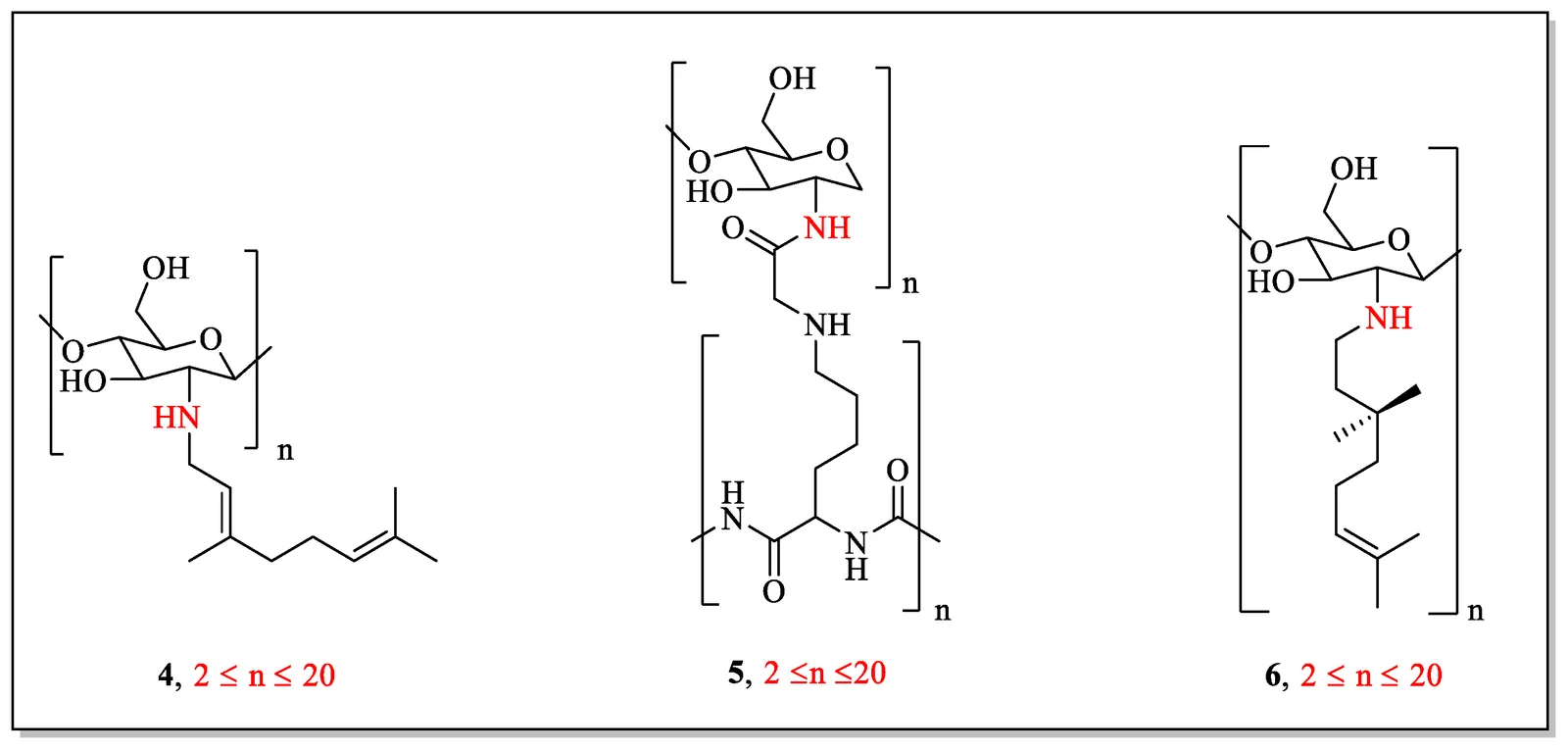
The chemical structures of compounds **4–6**.

Moreover, the MICs of COS-N-Ger (**4**, Figure 3) against *S. aureus* and *E. coli* were 0.5 and 1.0 mg/mL, respectively [58]. The oxygen radical absorbance capacity (ORAC) of LF-COS (**5**, Figure 3) reached 106.65 μmol Trolox per gram, and its ABTS cation radical scavenging activity was significantly higher than that of native lactoferrin [59]. The paw oedema inhibition rates of COS-g-Cit (**6**, Figure 3) with different degrees of substitution were 22.58, 29.03, and 25.81%, respectively [60].

### 2.5. Other Bioactivities

In addition to traditional medical applications, the potential of their derivatives in the field of plant pathology and metabolomics is gradually being explored. In agriculture, CT and its derivatives are regarded as pathogen-associated molecular patterns (PAMPs) that trigger plant innate immunity. Plants can activate defense signaling pathways by recognizing *N*-acetyl COS and induce the synthesis of phytoalexins and defense enzymes [31]. The degree of polymerization and acetylation mode of *N*-acetyl COS are the key factors that determine their effectiveness as plant elicitors [61]. Different from the non-specific mechanism of antibacterial activity, pattern of acetylation (PA) has a decisive regulatory effect on the immunomodulatory activity mediated by CS and COS receptors. The plant pattern recognition receptor CERK1 and the mammalian innate immune receptor TLR2 have a strict subsite binding preference for the acetylated sequence of the ligand. However, the samples of random PA can only trigger a very low level of immune response due to the lack of abundance of highly acetylated sequences. This rule not only clarifies the structure–activity nature of CS as a plant immune elicitor but also provides a theoretical basis for its precise application in the field of biomedicine-agricultural crop disease-resistant immune activation [17,21].

The value of CT derivatives as functional dietary supplements lies in their dual regulation of the intestinal–metabolic axis. COS with high content of acetylated residues can increase Lactobacillus/Enterococcus, showing excellent prebiotic effects [62]. At the host metabolic level, Zhen et al. confirmed that ultra-low molecular weight *N*-acetyl COS (DP < 3) has significant lipid-lowering activity. In the ApoE^-/-^ atherosclerotic mouse model, these small molecules effectively delayed the formation of arterial plaques by improving lipid metabolism disorders. This result indicates that CT-derived bioactive molecules are expected to cross the intestinal barrier or exert a systemic protective effect on the host’s cardiovascular health by regulating intestinal flora metabolites [63]. In the field of biomedicine, these CT scaffolds with natural configuration exhibit excellent biocompatibility, excellent structural integrity and low immunogenicity, and they have become ideal candidate materials for tissue engineering and regenerative medicine. As a targeted carrier, it is expected to efficiently encapsulate marine natural products or related chemically modified derivatives, showing broad application prospects in the targeted prevention and treatment of major pathogenic bacteria, pathogenic fungi, parasites and viral infections in aquaculture and veterinary fields [15,18].

In addition to tissue engineering and wound repair, COS-based nanomaterials also show good application prospects in dental restoration, periodontal antibacterial and other stomatology fields [64]. Beyond clinical medical applications, sponge CT also plays a crucial role in the rapidly emerging field of extreme biomimetics. Thanks to its excellent heat resistance and chemical stability, the 3D CT scaffold can withstand extreme hydro-thermal conditions. This property enables it to be used as a universal and thermally stable template for the deposition of a variety of inorganic compounds and in situ synthesis of new organic-inorganic composite biomaterials. These biomineralized composites exhibit excellent properties in electricity, catalysis and biosensing, which profoundly highlights the great potential of biomimetic materials derived from the oldest multicellular organisms in the ocean [8].

## 3. Structural Modifications of Chitin, Chitosan and Chitooligosaccharide

Although natural CT is rich in reserves, its high crystallinity and poor solubility (only soluble in strong acids or specific solvents such as DMAC/LiCl) are caused by its stubborn hydrogen bond network, seriously restricting its application range. In contrast, low-molecular-weight COS has solved the solubility problem, but the single structure limits its effectiveness in complex biological environments. The synthesis of *N*-acetylglucosamine derivatives can further expand their functional diversity through molecular modification [65]. Modifications of the C2-amino, C3-hydroxy, and C6-hydroxy groups on the CT/CS/COS skeleton were conducted.

### 3.1. Structural Modifications of Chitin and Chitosan

**Scheme 1:**
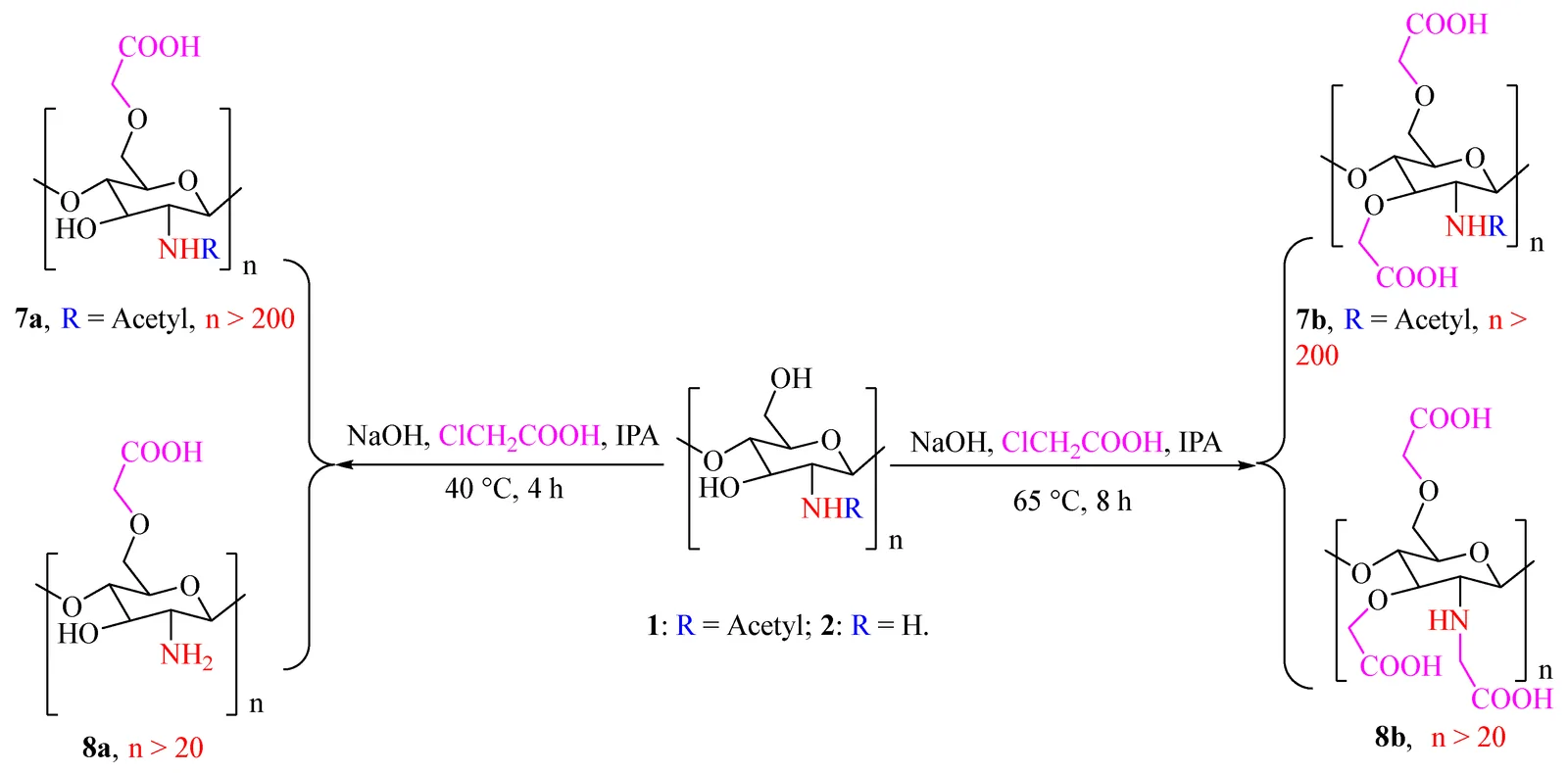
Synthesis of compounds **7a,b** and **8a,b**.

As shown in Scheme 1, Islam et al. synthesized a series of water-soluble carboxymethyl CT and CS derivatives (**7a,b** and **8a,b**) from chitin (**1**) and chitosan (**2**). Their antibacterial activities were evaluated against five Gram-negative bacteria (*Shigella flexneri*, *Enterococcus faecalis*, *Pseudomonas aeruginosa*, *Klebsiella pneumoniae*, and *Vibrio parahaemolyticus*) and three Gram-positive bacteria (*S. aureus*, *B. subtilis*, and *Bacillus cereus*). The results indicated that all synthesized compounds displayed moderate to great antibacterial activity against the above pathogenic bacterial strains [66].

**Scheme 2:**
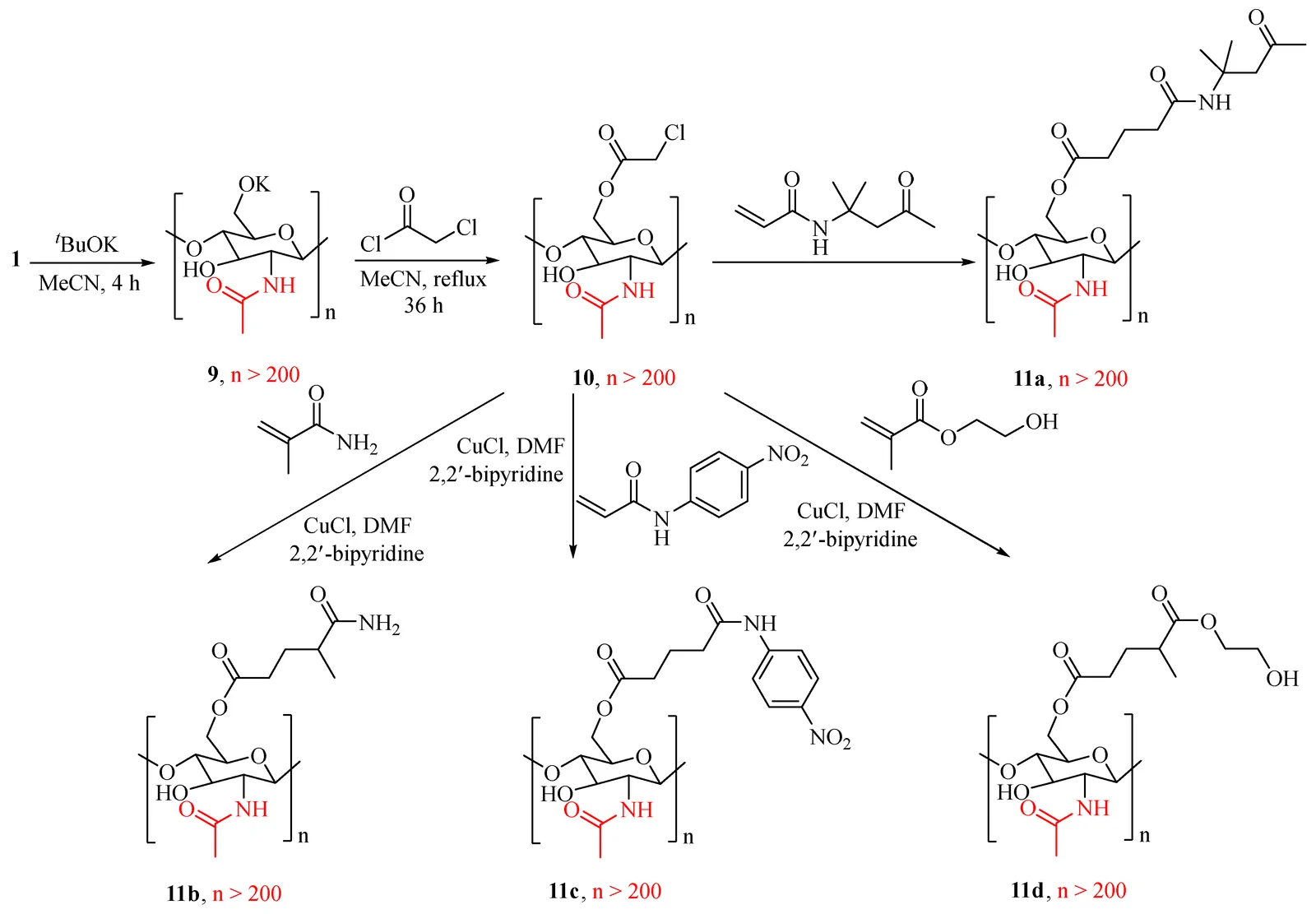
Synthesis of compounds **11a–11d**.

As illustrated in Scheme 2, firstly, compound **1** was deprotonated with potassium-*tert*-butoxide to afford compound **9**, which then reacted with chloroacetyl chloride under reflux in acetonitrile to yield intermediate **10**. Finally, compound **10** reacting with distinct monomers such as DAAM, MAM, NPA, or HEMA yielded target compounds **11a–11d**, respectively. The bioassay results demonstrated that compounds **10** and **11a–11d** exhibited dual potent antioxidant and anti-tumor activities. In particular, compound **10** showed the highest DPPH scavenging activity among all tested compounds, achieving a scavenging rate of 56.95%. It also displayed the most potent antitumor activity, reducing L1210 cell viability to 8.00 ± 0.91% after 24 h at 2.5 mg/mL [44].

**Scheme 3:**
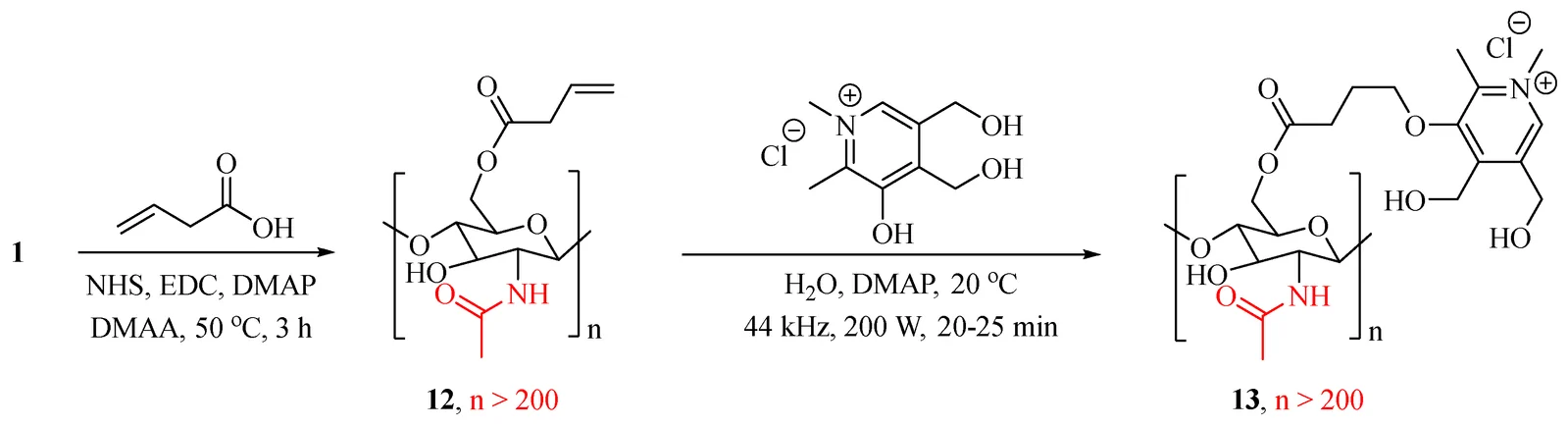
Synthesis of compounds **12** and **13**.

As described in Scheme 3, Egorov et al. pioneered the application of ultrasonic phenol-ene coupling as a polymer analogous transformation. Briefly, alkene-functionalized CT derivative **12** reacted with *N*-methyl pyridoxine chloride under the sonicated condition at 44 kHz and 200 W for 20–25 min to efficiently give the polymer **13**. Notably, compound **13** exhibited the pronounced antibacterial activity in rats without observable toxicity in vivo, which has potential for clinical applications [67].

### 3.2. Structural Modifications of Chitooligosaccharide

**Scheme 4:**
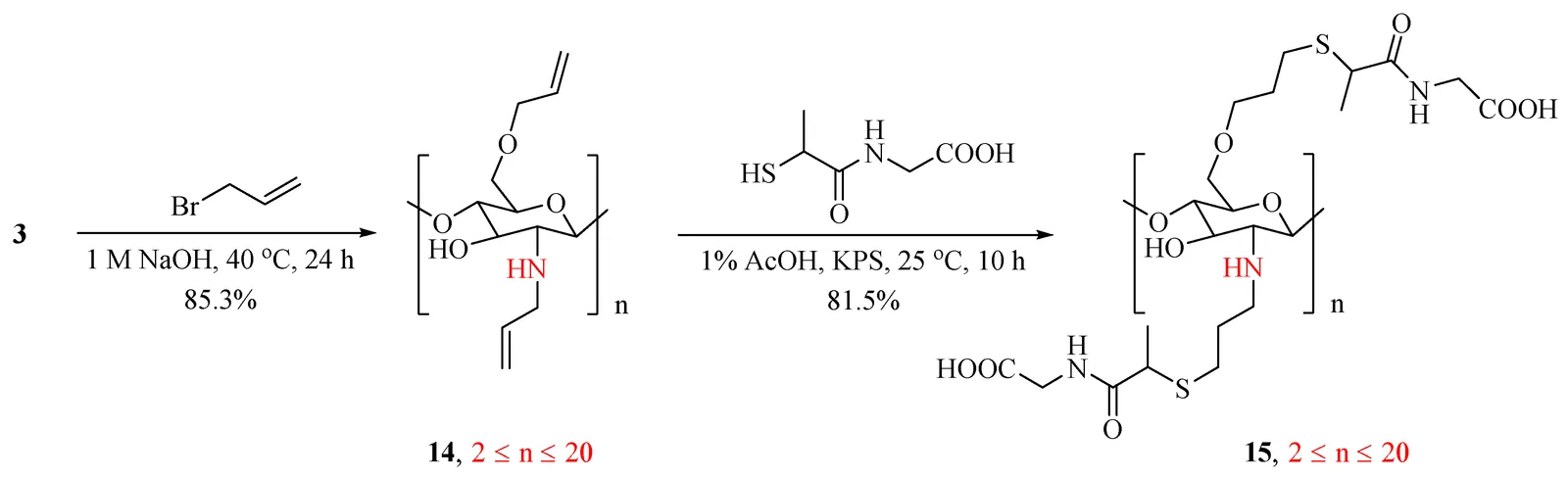
Synthesis of compounds **14** and **15**.

As depicted in Scheme 4, firstly, COS (**3**) was etherified with allyl bromide under alkaline conditions to afford compound **14**. Subsequently, it underwent a thiol–ene reaction with tiopronin to yield the target compound **15**. Compound **15** exhibited more potent antioxidant activities than its precursor **3**, and its IC_50_ values for scavenging DPPH, ABTS^+^ and OH were 0.31, 0.39 and 0.73 mg/mL, respectively, while the corresponding IC_50_ values of compound **3** were 0.66, 2.89 and 1.41 mg/mL, respectively. Additionally, compound **15** also showed a better antibacterial effect than **3** against pathogenic bacterial strains, without observable toxicity to RAW264.7 macrophages and MRC-5 human lung cells, suggesting its promising potential in therapeutic applications [68].

**Scheme 5:**
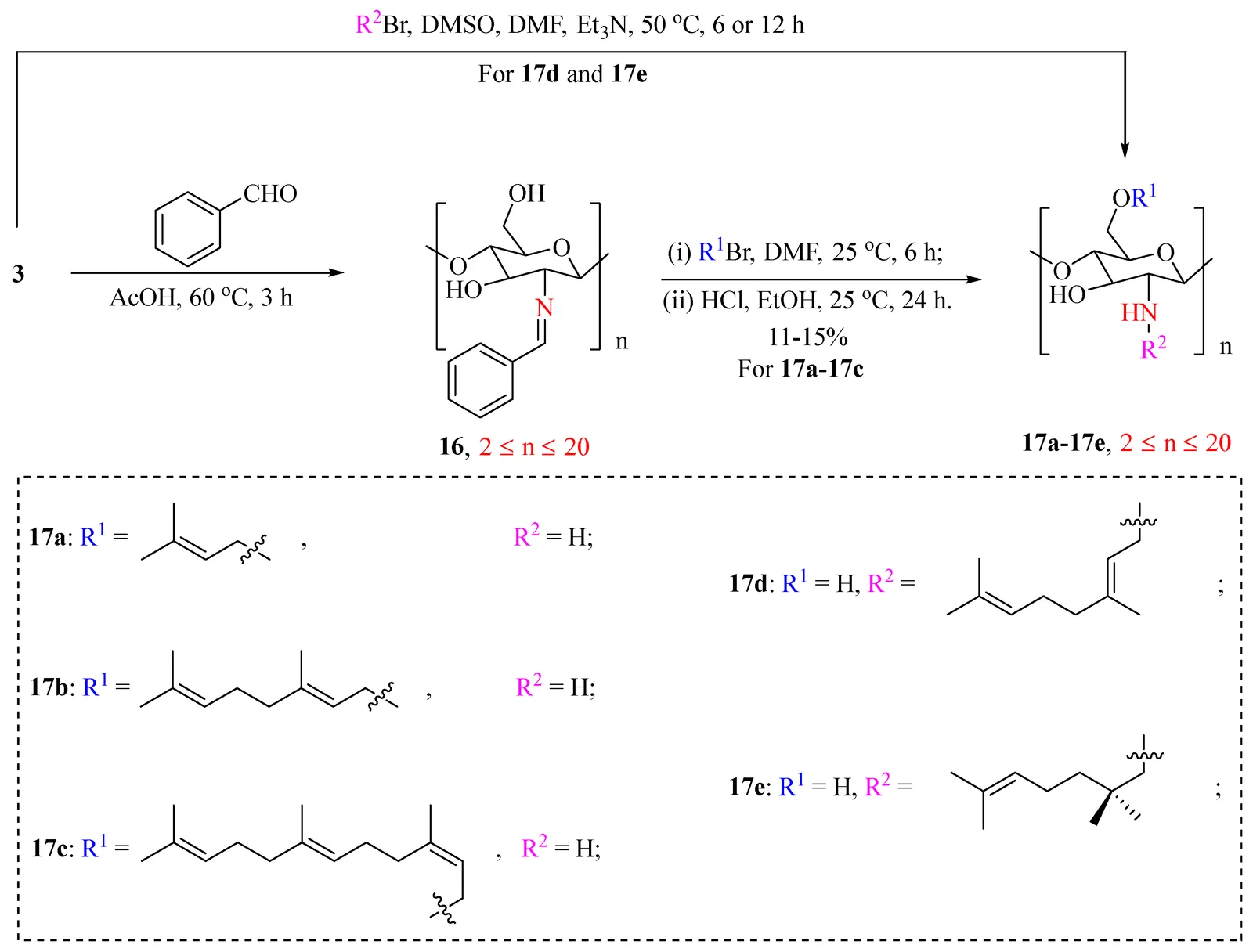
Synthesis of compounds **17a–17e**.

As described in Scheme 5, Yue et al. designed and synthesized a novel class of COS derivatives containing terpenol moieties (**17a–17c**). These compounds possessed excellent solubility and displayed more sensitive in vitro antibacterial activity against *S. aureus* than *E. coli*, revealing the pronounced potential applications as natural antibacterial agents [69]. Additionally, the COS derivative bearing a geraniol moiety (**17d**) was synthesized and evaluated its antibacterial activity. The results showed that compound **17d** presented a significant inhibition effect on *S. aureus* than *E. coli* [58]. It is worth noting that compound **17e** was obtained under the similar reaction conditions, which can drastically reduce the paw swelling, showing significant anti-inflammatory activity in vivo. The mechanism analyses indicated that compound **17e** can suppress the expression level of TNF-α via promoting the secretion of IL-4 and IL-10. Moreover, Western blot results proved that it can repress the NF-*κ*B signaling pathway through inhibiting the phosphorylation of p65, IKBα and IKKβ [60].

**Scheme 6:**
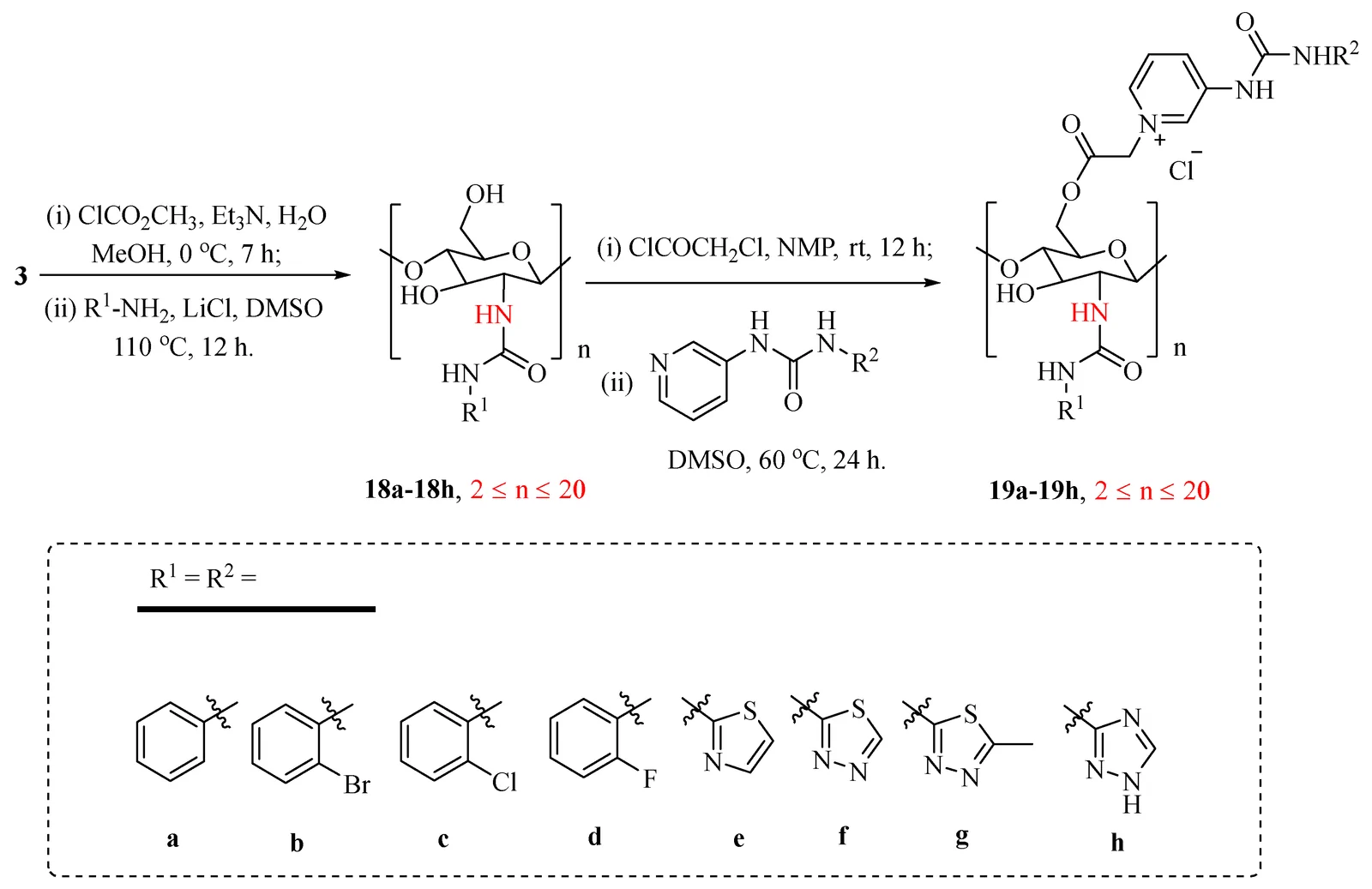
Synthesis of compounds **18a–18h** and **19a–19h**.

As depicted in Scheme 6, Zhang et al. prepared a series of 2-urea-COS (**18a–18h**) and 2,6-diurea-COS derivatives (**19a–19h**) as antifungal and antioxidant agents. Against *Botrytis cinerea*, compounds **19d** and **19h** showed the most potent antifungal properties, with inhibitory values of 66.67% and 67.68%, respectively. Moreover, the DPPH-radical scavenging results indicated that the scavenging rates of most derivatives were over 90% at 1.6 mg/mL. Notably, when the tested concentration was less than 500 μg/mL, the cytotoxic effects of all derivatives were weak and the cell viabilities can exceed 80%. Therefore, some 2,6-diurea-COS derivatives (**19a–19h**) can be considered as ideal medicinal biomaterials [70].

**Scheme 7:**
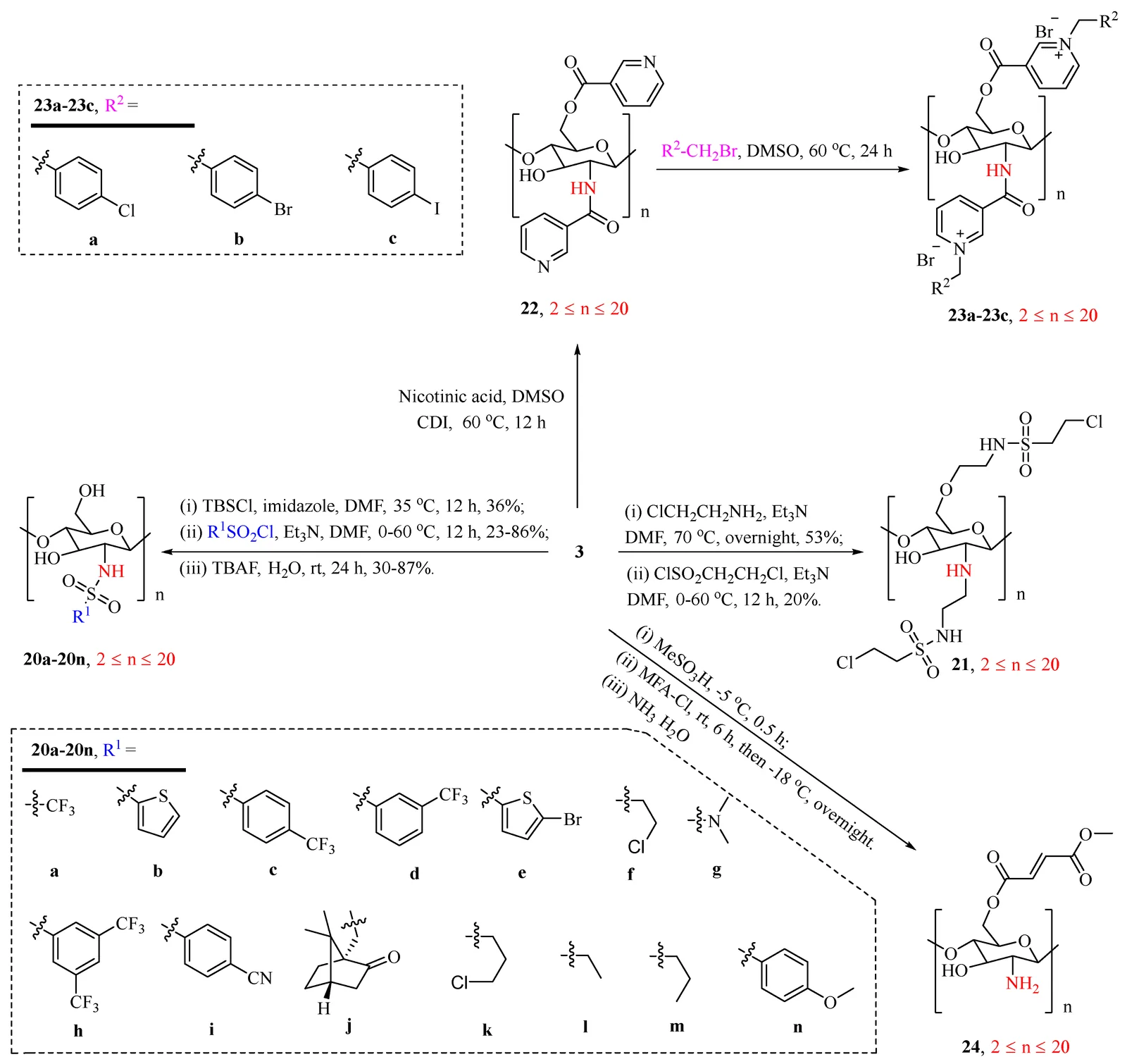
Synthesis of compounds **20–24**.

As described in Scheme 7, Fan et al. synthesized a series of COS sulfonamide derivatives (**20a–20n** and **21**) through hydroxylated protection and deprotection reactions. Among them, compounds **20f** and **20k–20m** exhibited high mortality (>98% at 1 mg/mL) against *Meloidogyne incognita*. In particular, derivative **20f** induced vacuole-like structures in the middle body and tail regions of the nematode, significantly suppressed egg hatching, and exhibited good control effects and low phytotoxicity in vivo [71].

Lin et al. firstly synthesized the COS-niacin acid intermediate (**22**) by using CDI as a catalyst. Then, cationic target compounds (**23a–23c**) were obtained by further nucleophilic reaction with halogenated benzyl bromide. Against *Fusarium oxysporum*, compound **23a** displayed the most significant antifungal activity, which was close to the commercial fungicide carbendazim. These results proved that it could be used as ideal fungicidal biomaterials [72].

Zhao et al. successfully introduced the MFA moiety at the hydroxyl group of COS to afford the target compound **24**, which exhibited more potent inhibitory effects against *S. aureus* and *E. coli* than its precursor **3** [73].

## 4. Mechanisms of Action

The analysis of molecular targets and signal transduction networks of chitin and its derivatives is a prerequisite for their precise application. Based on the existing omics and molecular biology evidence, their mechanisms of action can be explained from membrane interaction, redox balance, and intracellular signal regulation.

**Figure 4:**
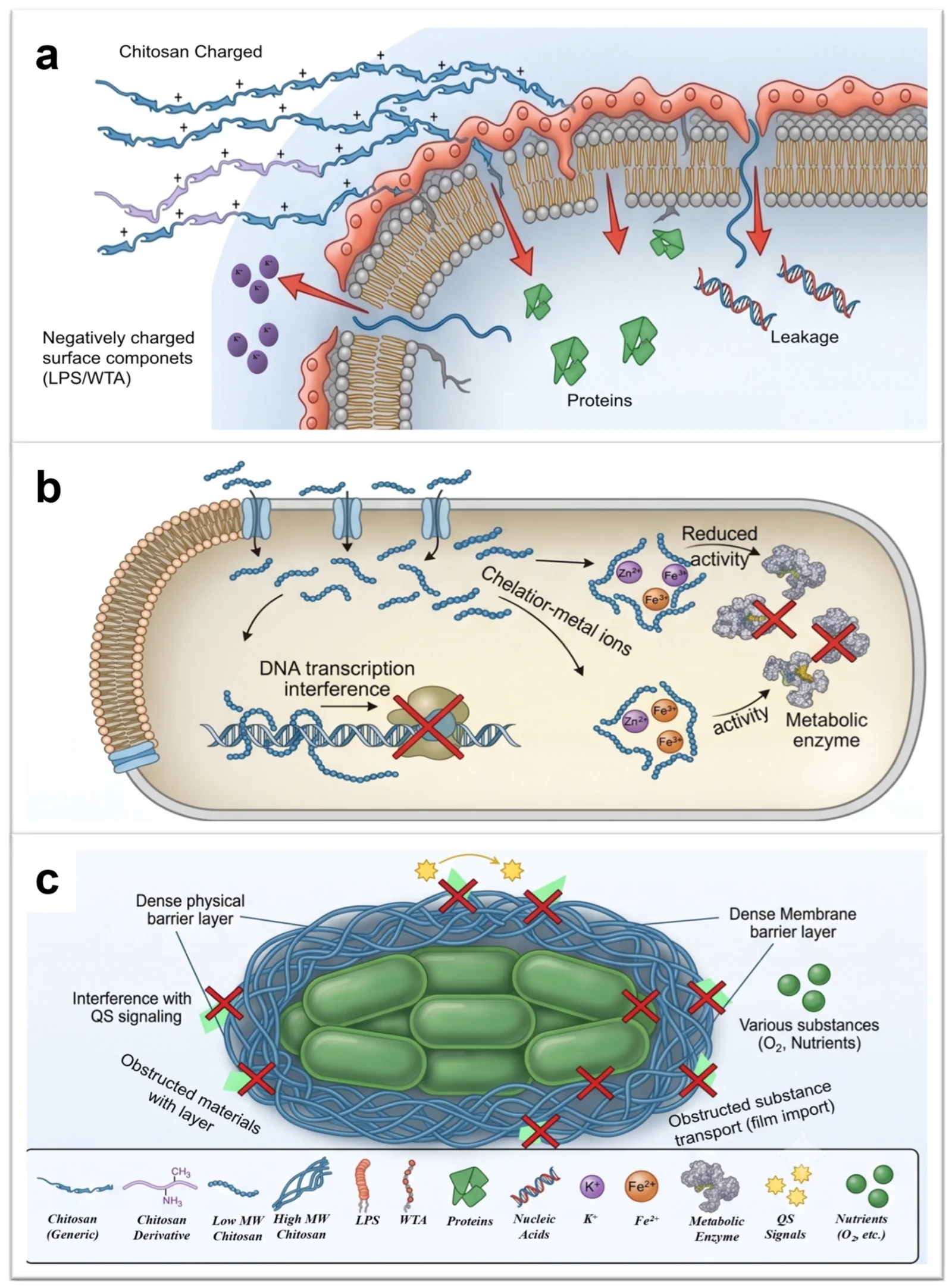
Schematic representation of antimicrobial mechanisms of chitosan and its derivatives. (**a**) The combination of positively charged chitosan with negatively charged bacterial surface leads to membrane rupture and leakage of contents. (**b**) Low-molecular-weight chitosan penetrates cell membranes, interferes with DNA transcription and chelates metal ions in cells. (**c**) High-molecular-weight chitosan forms a dense membrane in vitro to block substance transport and interfere with quorum sensing (QS) signals.

As demonstrated in Figure 4, since its first report in 1979, the broad-spectrum antibacterial activity of chitosan has been proven to be the result of multi-mechanism synergy and is strongly influenced by its own molecular weight [74]. The main bactericidal effect of chitosan comes from the electrostatic interaction on the surface of bacteria. The protonated amino group (-NH_3_^+^) on the main chain of chitosan can strongly bind to the negatively charged components of microbial cell walls: lipopolysaccharides in Gram-negative bacteria and teichoic acid in Gram-positive bacteria. This binding will structurally disintegrate the stability of the cell membrane, irreversibly change the permeability of the cell membrane, and ultimately cause the immediate rupture of the cell membrane. Intracellular potassium ions (K^+^), proteins, nucleic acids and other key active substances were leaked to the outside of the cell, and the cells were rapidly apoptotic [20,37].

Due to their smaller size, low-molecular-weight chitosan chains can readily cross the compromised cell membrane to exert intracellular toxicity. Once inside the cytoplasm, they interfere with genetic expression by directly binding to DNA, which physically obstructs the transcription process. Simultaneously, the strong chelating ability of these chains allows them to sequester trace metal ions (such as Zn^2+^ and Fe^3+^) that are vital for microbial survival. The depletion of these cofactors inactivates key metabolic enzymes, thereby halting essential intracellular metabolic processes [40]. Conversely, high molecular weight chitosan predominantly acts in the extracellular environment. Its longer polymer chains entangle to form a dense, physical barrier layer encapsulating the bacterial cells. This membrane-like matrix restricts material exchange, effectively starving the bacteria by blocking the uptake of essential environmental oxygen and nutrients. Beyond physical isolation, this dense network also intercepts and interferes with the transmission of quorum-sensing (QS) signaling molecules, fundamentally disrupting bacterial communication and preventing the maturation of protective biofilms [23,40]. Chitooligosaccharide (COS) also has a unique autolysis induction mechanism. COS could induce the abnormal increase of chitinase activity in fungal cells, break the dynamic balance of chitin synthesis and degradation, and lead to the distortion of mycelial morphology and the disintegration of cell wall structure [39].

Chitosan and its derivatives possess well-documented reactive oxygen species (ROS) scavenging capabilities, primarily driven by the abundant hydroxyl (-OH) and amino (-NH_2_) groups along the polymer backbone. These functional groups act as effective hydrogen donors, readily neutralizing unstable free radicals [4]. In particular, some studies indicate that a lower MW fraction yields superior scavenging efficiency, and from our own perspectives, shorter chains generally exhibit greater conformational flexibility, which facilitates molecular stretching and maximizes the exposure of active chemical groups. Yet, as chain length varies, shifts in the intra- and intermolecular hydrogen bonding networks can fundamentally alter the local reactivity of these active sites. Consequently, the spatial accessibility and overall density of the functional groups—rather than the pure molecular weight alone—likely serve as the true underlying determinants of antioxidant efficacy [46,75,76]. While inflammation serves as a fundamental physiological defense against tissue damage and invading pathogens [17], its overactivation frequently leads to pathology. Lipopolysaccharide (LPS), a potent endotoxin, drives this excessive response by triggering the massive release of cytokines and inflammatory mediators across macrophages, monocytes, and mast cells, acting as a primary catalyst in the progression of various inflammatory diseases [77].

**Figure 5:**
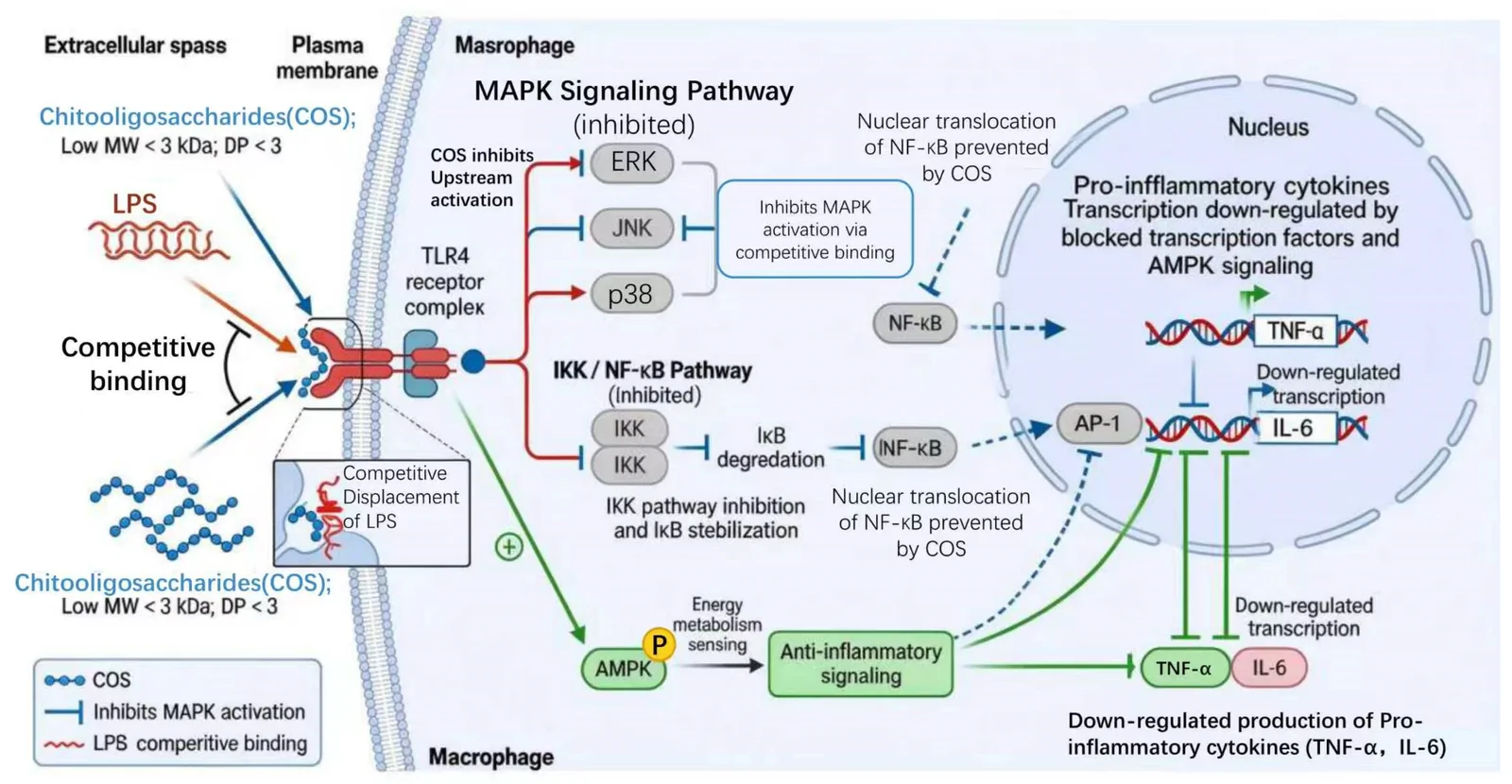
A schematic diagram of the anti-inflammatory molecular mechanism of COS in macrophages. COS blocks the upstream inflammatory signal transduction by competitively binding to TLR4 receptor complex with LPS. This binding not only inhibited the activation of MAPK (ERK, JNK, p38) and IKK/NF-*κ*B signaling pathways, and prevented the nuclear translocation of NF-*κ*B and AP-1, but also activated the AMPK-mediated energy metabolism sensing pathway. These synergistic effects ultimately significantly down-regulated the transcription and expression of pro-inflammatory cytokines (TNF-α and IL-6).

As displayed in Figure 5, COS offers a promising countermeasure by actively reshaping this inflammatory microenvironment. COS competitively binds to the TLR4 receptor complex, effectively neutralizing LPS-induced signaling at its source. This critical upstream blockade suppresses the subsequent phosphorylation and activation of both the MAPK (ERK, JNK, p38) and IKK/NF-*κ*B cascades. Consequently, the nuclear translocation of key transcription factors (namely AP-1 and NF-*κ*B) is severely restricted, and the O-GlcNAcylation modification of NF-*κ*B is also inhibited, decisively down-regulating the transcription of pro-inflammatory cytokines such as TNF-α and IL-6 [78,79]. Concurrently, COS reinforces this immunosuppression by activating the AMPK-mediated energy sensing pathway [29].

From a structure–activity perspective, the efficacy of this immunomodulation is highly size-dependent. COS fractions characterized by a low molecular weight (<10 kDa) and a low degree of polymerization (DP < 8) exhibit vastly superior anti-inflammatory capabilities. This enhanced bioactivity is directly attributed to their optimized transmembrane transport efficiency, allowing them to rapidly penetrate the cell and intercept intracellular inflammatory signals [30,80].

One of the core objectives of oncology research is to redefine cancer as a clinically controllable disease. In the current revolutionary medical model, a variety of innovative treatment strategies have been proposed, which can significantly enhance the body’s anti-tumor immune response [32,81,82]. COS has shown intervention potential at all stages of tumor development (anticancer pleiotropy) [30]. COS can induce cell cycle arrest and induce cancer cell death by activating the Caspase-9-dependent mitochondrial apoptosis pathway [55]. Meanwhile, COS-Se can repress the growth of gastric adenocarcinoma through reducing levels of CD34, VEGF and MMP-9 [57].

## 5. Structure–Activity Relationships of Chitin, Chitosan, Chitooligosaccharide, and Their Derivatives

**Figure 6:**
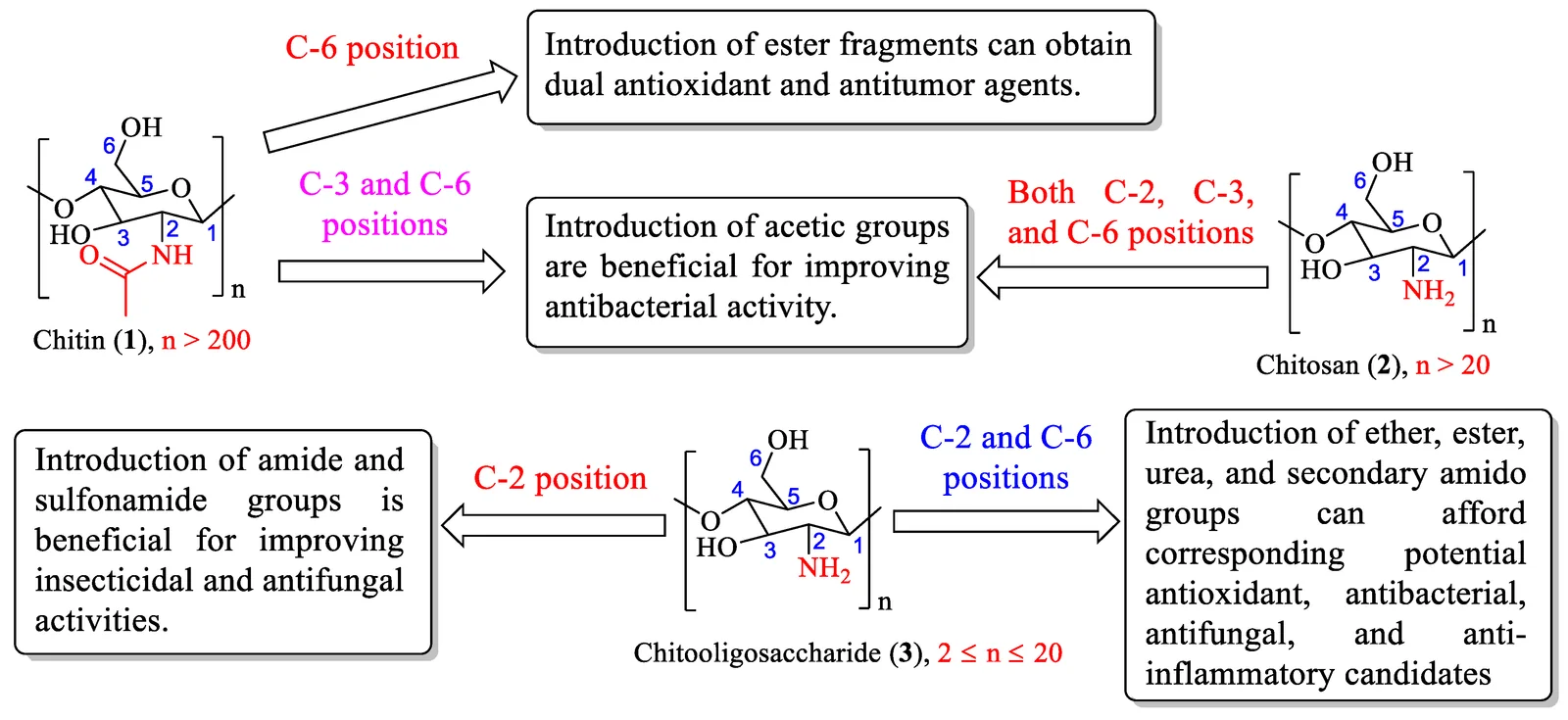
The structure–activity relationships of chitin (**1**), chitosan (**2**), chitooligosaccharide (**3**) and their derivatives.

The SAR analysis of chitin (**1**), chitosan (**2**), chitooligosaccharide (**3**) and their derivatives is described in Figure 6. The detailed descriptions are as follows: (i) For synthetic chitin derivatives, introduction of acetic groups at C-3 and C-6 hydroxy positions can give potent antibacterial candidates [66]. Moreover, the presence of ester groups at the C-6 position also help to improve antibacterial activity and may obtain dual antioxidant and anti-tumor agents [44,67]. (ii) For chitosan derivatives, introduction of acetic groups at both C-2, C-3, and C-6 hydroxy positions also can afford antibacterial compounds [66]. (iii) For chitooligosaccharide derivatives, introduction of ether, ester, and secondary amido groups at the C-2 and C-6 positions can afford potential antioxidant, antibacterial, and anti-inflammatory derivatives [58,60,68,69,73]. Additionally, it is worth noting that 2,6-diurea-COS derivatives show pronounced antifungal property against *B. cinerea* and high DPPH-radical scavenging rates, so they could be considered as ideal medicinal biomaterials [70]. The introduction of amide and sulfonamide groups at the C-2 position is beneficial for improving agricultural activities, such as insecticidal and antifungal effects [71,72].

## 6. Conclusions

In recent years, research and development of marine-derived extracts, such as CT (**1**), CS (**2**), and COS (**3**), have been steadily improving. The commercialization of these ideal candidate materials should capitalize on their benefits and prioritize sustainability. In the future, they will play a significant role in the application of medicinal biomaterials, improving the development of medical devices and human health levels. In this review, we have summarized the research progress from 2020 to 2025 on biological activities, structural modifications, and SARs of compounds **1–3** and their derivatives, in addition to their mechanisms of action. It is prospective that this paper can offer much information and essential proposals for further structural modifications and development of novel derivatives as potent marine-derived drugs and biomaterials for improving human health levels.

## Figures and Tables

**Table 1:** An overview of the biological activities of synthetic COS derivatives.

Biological Activities	COS Derivatives	COS Characteristics *	Effects	Ref
Anti-tumor activity	AZD-PLGA-COS	DP = 2–10 DD = 95%	Enhancing antitumor efficacy of AZD9291 (osimertinib) by promoting apoptosis via the p-EGFR/PARP/Bak/caspase-9/Bax/Bcl-2 pathway.	[55]
	COS-Se	MW = 1268 Da DD = 91.3%	Inhibiting tumor cell proliferation and migration; suppressing angiogenesis via down-regulating CD34, VEGF and MMP-9; enhancing immune function through selenium supplementation.	[57]
Antibacterial activity	COS-N-Ger (**4**)	MW = 1500 Da DD = 95% DS = 0.260	Disrupting bacterial cell membrane integrity, increasing permeability and causing leakage of intracellular contents.	[58]
Antioxidant activity	LF-COS (**5**)	MW = 1500 Da	Exhibiting strong scavenging capacity against DPPH, ABTS, hydroxyl radicals and superoxide radicals, and regulating the gene expression of antioxidant enzymes.	[59]
Anti-inflammatory activity	COS-g-Cit (**6**)	MW = 1000 Da DD = 95% DS = 0.65	Suppressing the expression level of TNF-α via promoting the secretion of IL-4 and IL-10; repressing the NF-*κ*B signaling pathway through inhibiting the phosphorylation of p65, IKBα and IKKβ.	[60]

* MW: molecular weight; DP: degree of polymerization; DD: degree of deacetylation (%); DS: degree of substitution (%).

## Data Availability

No new data were created or analyzed in this study. Data sharing is not applicable to this article.

## Abbreviations

AcOH	Acetic acid
BAAM	N-*tert*-Butyl acrylamide
CDI	*N,N*-Carbonyldiimidazole
DA	Degree of acetylation
DMAA	*N,N*-Dimethylacetoacetamide
DMAC	*N,N*-Dimethylacetamide
DMAP	4-Dimethylaminopyridine
DMF	*N,N*-Dimethylformamide
DMSO	Dimethyl sulfoxide
DP	Degree of polymerization
DPPH	2,2-Diphenyl-1-picrylhydrazyl
DS	Degree of substitution
EDC	1-Ethyl-3-(3-dimethylaminopropyl) carbodiimide
EtOH	Ethanol
HEMA	2-Hydroxyethyl methacrylate
IC_50_	Half-inhibitory concentration
IPA	Isopropyl alcohol
KPS	Potassium persulfate
MAM	Meth acrylamide
MBA	*N,N*-Methylenebisacrylamide
MIC	Minimum inhibitory concentration
MW	Molecular weight
NF-*κ*B	Nuclear factor-*κ*B
NHS	N-Hydroxy succinimide
NIP	N-Isopropylacrylamide
Nrf2	Nuclear factor erythroid 2-related factor 2
NSAIDs	Non-steroidal anti-inflammatory drugs
ROS	Reactive oxygen species
SAR	Structure–activity relationship
TBAF	Tetrabutylammonium fluoride
TBSCI	*Tert*-Butyldimethylsilyl chloride
TEA	Triethylamine
VEGF	Vascular endothelial growth factor
WTA	Wall teichoic acid
